# Single neuron recordings of bilinguals performing in a continuous recognition memory task

**DOI:** 10.1371/journal.pone.0181850

**Published:** 2017-08-23

**Authors:** Erika K. Hussey, Kiel Christianson, David M. Treiman, Kris A. Smith, Peter N. Steinmetz

**Affiliations:** 1 Cognitive Science Team, U.S. Army Natick Soldier Research, Development and Engineering Center, Natick, Massachusetts, United States of America; 2 Center for Applied Brain and Cognitive Sciences, Medford, Massachusetts, United States of America; 3 Beckman Institute for Advanced Science and Technology, University of Illinois, Urbana, Illinois, United States of America; 4 Department of Educational Psychology, University of Illinois, Champaign, Illinois, United States of America; 5 Department of Neurology, Barrow Neurological Institute, Phoenix, Arizona, United States of America; 6 Department of Neurosurgery, Barrow Neurological Institute, Phoenix, Arizona, United States of America; 7 Nakamoto Brain Research Institute, Tempe, Arizona, United States of America; University of Hyderabad, INDIA

## Abstract

We report the results of a bilingual continuous recognition memory task during which single- and multi-neuron activity was recorded in human subjects with intracranial microwire implants. Subjects (n = 5) were right-handed Spanish-English bilinguals who were undergoing evaluation prior to surgery for severe epilepsy. Subjects were presented with Spanish and English words and the task was to determine whether any given word had been seen earlier in the testing session, irrespective of the language in which it had appeared. Recordings in the left and right hippocampus revealed notable laterality, whereby both Spanish and English items that had been seen previously in the other language (switch trials) triggered increased neural firing in the left hippocampus. Items that had been seen previously in the same language (repeat trials) triggered increased neural firings in the right hippocampus. These results are consistent with theories that propose roles of both the left- and right-hemisphere in real-time linguistic processing. Importantly, this experiment presents the first instance of intracranial recordings in bilinguals performing a task with switching demands.

## Introduction

Language has been the prime example of lateralization of cognitive function ever since Broca [1] and Wernicke [2] presented case studies of patients who suffered localized left-hemisphere (LH) damage resulting in language-specific deficits. In recent decades, research using functional magnetic resonance imaging (fMRI; [3–7]), magnoencephalagraphy (MEG; [8–9]), positron emission tomography (PET; [10–12]), intracortical recordings [13], and event-related potentials (ERPs; [14–17]) suggests that language function is more distributed than Broca and Wernicke initially proposed. Language appears to operate via a complex network of interconnected brain regions spanning *both* hemispheres [18–20].

One framework that makes explicit predictions about global hemispheric differences in language processing is Federmeier’s PARLO model [15] (for another similar model, see [21]). According to this framework, when it comes to lexical processing, recording ERPs while presenting words in one half of the visual field (i.e., the right visual field) suggests that the LH operates over message-level meanings [22], allowing for more predictive, or "top-down," processing. Moreover, the LH seems to be responsible for selecting the meanings of words that are appropriate in the given context. In contrast, the right hemisphere (RH) seems to operate in a more "bottom-up" fashion, recognizing incoming words as they are encountered, operating over lower-level features, and retaining a more veridical representation of the input. Federmeier [15] reports results from a study by Metcalf et al. [23] in which split-brain patients in a lexical recognition memory task displayed a RH advantage in rejecting "lures" that were semantically similar to words that had been previously studied (cf. [24] with brain-intact participants). Federmeier points out that data such as these have been taken as evidence that the LH attends to "category invariant input features" (p. 499 in [15]), such as meaning, while the RH "processes and retains veridical, item-specific…and perhaps form-specific" representations of input (p. 499 in [15]). In addition to delineating hemispheric contributions during language processing, the PARLO model has been instrumental in distinguishing top-down and bottom-up processes during word learning [25] and non-literal sentence processing [26].

Suggestive new evidence, to be summarized below, about the functional role of the hippocampus in real time interpretation decisions further supports a more interconnected picture of language processing. Traditionally, the hippocampus has been linked to the formation of long-term declarative memory, including the acquisition, storage, and retrieval of declarative information [27]. These representations can be updated and consolidated over long periods of time to incorporate new sources of information [28–29].

Only recently has the hippocampus been examined as potentially having a role in real- time language processing [30–31], especially when comprehenders must flexibly update linguistic representations as they engage in activities like referential communication [32] and incremental sentence processing [33]. There is some evidence suggesting a central role of the hippocampus in the creation and integration of relational representations (i.e., input is bound with situation, context, speakers, and intentions), as well as the flexible expression of these representations [32]. Duff and Brown-Schmidt [32] point to studies of hippocampal amnesiacs and fMRI investigations of normal hippocampal activity, all of which suggest that "new hippocampus-dependent representations are available rapidly enough to influence ongoing processing when new information is perceived; old information is retrieved; and representations are held on-line to be evaluated, manipulated, integrated, and used in service of behavioral performance" (p. 5 of [32]).

Further insight into the role of the hippocampus in lexical access is provided by Hernandez [34], who measured hemodynamic changes in Spanish-English bilinguals as they named pictures in both their dominant (Spanish) and second (English) languages. Hernandez [34] observed an interesting lateralization difference, such that picture naming in Spanish generated larger changes in the left hippocampus, whereas picture naming in English generated larger changes in the right hippocampus. Hernandez cites related data by Whatmough and Chertow [35] that showed increased LH hemodynamic changes correlated with monolingual word retrieval, and increased RH changes with picture naming. Alongside Hernandez's results with bilinguals, the general conclusion is that picture naming in a bilingual's dominant language is more reliant on semantic memory, subserved primarily by the left hippocampus. Picture naming in the non-dominant language, on the other hand, is more reliant on recognition memory, subserved primarily by the right hippocampus.

The view outlined by Federmeier [15] that the LH operates over more abstract, semantic representations, and that the RH operates over more concrete, surface-level, veridical representations, is consistent with the left- vs. right-hippocampal data presented by Hernandez [34]. That is, accessing engrained, default linguistic information (e.g., from one’s native language) relies heavily on LH “top-down” resources, a process perhaps akin to activating a *type* of some semantic representation (i.e., a general abstraction of meaning). Accessing less-familiar representations (e.g., from one’s second language) seems to be supported by RH “bottom-up” resources, perhaps due associated with the activation of *tokens*, or specific instances that contain item- and context-unique features. Furthermore, this picture is largely consistent with a proposal by Jiang and Forster [36] about the lexical representations of unbalanced bilinguals, or individuals who have unequal usage and proficiency of their two languages. The authors elaborate on the Revised Hierarchical Model (RHM; [37–38]) to state that lexical items in the native language (L1) are represented in an abstract "lexical memory" (or a type), such that representations are contextually independent. Lexical items in the second language (L2), on the other hand, have context-bound representations. These “tokens” contain surface-level (form) representations, along with tags of when and where they were encountered. These veridical tokens are accessed for L2 words prior to activating links to L1 representations and, subsequently, the semantic “type” representations.

In light of Hernandez’s [34] results, the PARLO model [15], and Jiang and Forster’s [36] proposal, one might predict discriminatory roles of the left and right hippocampus during bilingual processing, especially when bilinguals are asked to operate over input that is presented alternately in both the L1 and L2.

### Motivation and hypotheses for the present study

Despite the mounting evidence for intra-hemispheric neural signatures of bilingual language processing (for ERPs see [15]; for MRI see [34–35; 39] for PET see, [40]), little work has examined this pattern with a method that offers *combined* spatial and temporal sensitivity. The research reported here addresses this issue by *directly* measuring single neuron activity using microwires implanted within candidate brain regions during a recognition memory task. The data were collected from bilingual Spanish-English speakers as they performed a task, which required them to process interleaved Spanish and English words. The goal was to determine if there is evidence at the level of individual neurons for theoretical claims about the lateralization of language processing (e.g., [15]) with special reference to the hippocampus and the nature of lexical access in bilinguals (e.g., [36–38]).

**Fig 1:**
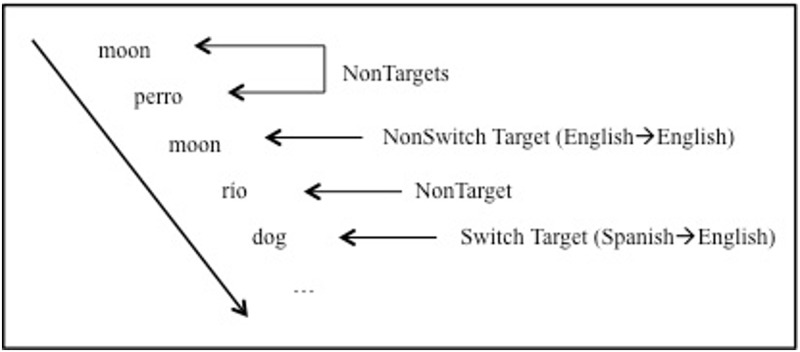
Bilingual continuous recognition memory task. Subjects are asked to indicate whenever a word repeats independently of the language in which it appears. NonTargets are new items presented for the first time and Targets are old items that repeat. NonSwitch Targets repeat in the same language (matching in form and meaning) and Switch Targets repeat in a different language (matching only in meaning).

We specifically tested whether hippocampal neurons differentiated tokens (surface form information) and types (semantic information) when bilinguals perform a continuous recognition memory task (CRM; [41–42]). In the bilingual CRM, participants are presented with a list of words from two languages, in this case English and Spanish. The participant's task is to determine whether each word that is presented has been seen before in the testing session or not (i.e., determine old/new status of words), *independent of* the language it appears (or appeared previously) in (Fig 1). In this way, the bilingual CRM engages both “top-down” and “bottom-up” processing: If the target word, e.g., *river*, has been seen previously in the list in English, a correct YES response can be provided by accessing “bottom-up” form information (tokens) or “top-down” meaning representations (types). If the target word, again *river*, has been seen previously in Spanish (i.e., *rio*), however, “bottom-up” processing, alone, cannot be used to answer correctly, as the exact surface form has not been encountered in the list. In this case, “top-down” processing must be used to access the semantic type that binds together both forms (*river* and *rio*) to determine that the correct answer is YES.

When the Jiang and Forster model [36] is set in the context of the more recent neurocognitive studies summarized by Federmeier [15] and the bilingual fMRI data of Hernandez [34], we can generate the following predictions. If the RH operates over more veridical, surface-level (token) representations, and if the right hippocampus is more active for “bottom-up” processing, then changes in neuron-level firing should be greater in the right hippocampus for targets that were previously seen in the same language (repeat trials). If the LH operates over more abstract, meaning-based (type) representations, and if the left hippocampus is more active for “top-down” processing, then changes in neural activity should be greater in the left hippocampus for targets that were previously seen in the different language (switch trials). To preview our results, we observe this pattern of lateralization, which constitutes the first report of single neuron activity in the brains of bilingual subjects as they switch between linguistic representations.

## Materials and methods

### Subjects and language proficiency

The participants were 5 patients with drug-resistant epilepsy requiring the implantation of depth electrodes (Ad-Tech Medical, Racine, WI) for clinical evaluation and consideration of possible surgical resection of their seizure focus. All patients provided informed written consent to participate in the research study. Consent was obtained by one of the authors (PNS) conducting a discussion with the patient of the risks and benefits of participating which was then documented using a written consent document. The procedures for obtaining consent and the consent document were reviewed and approved by the Institutional Review Board of St. Joseph’s Hospital and Medical Center (human subjects assurance #00001499). All patients were right-handed, Spanish-English bilinguals with mostly balanced levels of proficiency in both languages, as measured by their performance on Cloze tests administered in both languages. In each Cloze test, every seventh word was deleted, and subjects were instructed to fill in the missing word in each of resultant blanks (40 total blanks in the Cloze test in each language) (see Examples 1 and 2 below). We implemented two scoring methods. The first adopted a strict criterion, such that participants had to provide an exact match for the missing item in order to be scored as correct. The second used a relaxed coding standard by scoring any structurally and semantically valid options beyond just the expected word as correct. In both cases, a higher score corresponds to higher proficiency. Patient Cloze scores and demographics appear in Table 1. Using Cloze as an estimate of proficiency, two of five subjects were Spanish dominant and the remaining three were fairly balanced on this instrument. Note, however, that all subjects reported Spanish being their native language.

1. His mind wandered as he drove ______ *(past)* small farms and he began to ______ *(imagine)* living on his own piece of ______ *(land)* and becoming self-sufficient.
2. Es una del las tiendas más ______ *(ricas)* de las capital, y aunque hay ______ *(otros)* tiendas como la tienda de don Samuel, ______ *(ninguna)* es tan rica.

**Table 1:** Subject-level demographics and language proficiency.

			English Cloze		Spanish Cloze	
Subject	Age	Gender	Strict	Lax	Strict	Lax
S26	40	F	1	7	17	22
S37	31	F	15	26	23	32
S40	43	F	12	24	18	25
S49	49	M	22	24	26	36
S50	28	F	27	35	18	36

Spanish and English proficiency was measured using two scoring systems, strict and lax (see text).

### Bilingual continuous recognition memory task

During the bilingual CRM task, participants were instructed to judge semantic repetitions over the course of the experimental session. They were asked to provide a ‘no’ response to any items judged as a new and unseen and ‘yes’ response to any old items that repeated, regardless of the language in which the word was presented. There were 150 items, each one consisting of a Spanish-English translation pair. Thus each participant saw 300 experimental words (targets) interspersed with 60 Spanish and 60 English fillers that were not paired with translation equivalents. The use of filler words and a pseudo-random order of presentation was designed to ensure that the odds of a word being new on any given trial were 50% (for most participants, the design was unbalanced because the experiment was often terminated early due to clinical constraints). As illustrated in Fig 1, the first presentation of each member of the translation pair (to which participants should have responded "No"; **non-target**) was followed, after 0–31 intervening words, by either another presentation of the same word (**target**) in the same language (**non-switch**) or by its translation equivalent in the opposite language from the first presentation (**switch**). Half of all eventual targets (75) appeared first in English, and half (75) in Spanish. Half of these targets (give or take one item, due to the odd number) repeated in the same language (e.g., *shoe* appearing after *shoe*) and roughly half repeated in the opposite language (e.g., *shoe* appearing after *zapato*). On a single trial of the task, participants saw a fixation cross for 400–600 ms, followed by the to-be-judged word for 1000 ms, followed by a question mark, which appeared until a response was made or until 2000 ms had elapsed. The lag separating a repeating item from its initial presentation ranged from 1 (immediate repeat) up to 32 intervening items, though we did not examine lag as a factor in our analyses due to the limited number of observations at each lag.

#### Word set

The stimulus words included Spanish and English adjectives, verbs, and nouns (50 from each word class) that were drawn from the LexEsp corpus for Spanish words [43] and the COCA corpus for English words [44]. Stimuli were carefully selected by the second author and a fluent Spanish-speaking linguist according to three criteria. First, the items were matched pairwise in log frequency based on frequency per million in their respective corpora (English: *M* = 1.947, Spanish: *M* = 1.943) and string length (English: *M* = 5.68, Spanish: *M* = 6.38). Second, items were selected to control for number of senses in both languages, i.e., as much as possible, each item has one, or one extremely dominant, sense. For example, words with multiple senses like "bank" (bank of a river, bank for money, bank as a kind of shot) were excluded. Finally, items were selected that have, as much as possible, only one direct or extremely frequent translation in the corresponding language.

Valence and arousal norms for 141 or the 150 English stimuli used here are provided in Table 2 [45]. Mean valence (~5.2) and arousal (~4.3) norms qualify as neutral and moderately low, respectively. Valence and arousal norms for 30 or the 150 Spanish stimuli (nouns and adjectives only) used here are provided [46] in Table 3. Mean valence (~4.6) and arousal (~5.48) norms qualify as neutral for both ratings. The slightly higher overall arousal rates for Spanish words compared to English words is consistent with the full Spanish data set reported in [46] as compared against their English counterparts in [47].

**Table 2:** Valence and arousal for English word categories (141 out of 150).

English	Valence			Arousal		
Word Type	Mean	SD	Range	Mean	SD	Range
Noun	5.54	1.11	2.74–7.88	4.00	0.99	2.40–6.05
Verb	5.16	1.53	1.96–7.89	4.42	1.10	2.24–7.10
Adjective	4.95	1.75	2.1–7.59	4.64	1.02	2.25–6.95
All Items	5.21	1.50	1.96–7.89	4.35	1.07	2.24–7.10

**Table 3:** Valence and arousal for Spanish word categories (30 out of 150).

Spanish	Valence			Arousal		
Word Type	Mean	SD	Range	Mean	SD	Range
Noun	5.39	1.83	2.06–7.20	5.83	2.03	4.03–7.38
Verb	No data	No data	No data	No data	No data	No data
Adjective	4.16	1.61	1.61–7.79	5.40	2.00	4.16–6.93
All Items	4.56	1.68	1.61–7.79	5.48	2.01	4.03–7.38

SD = standard deviation

### Behavioral response measures

We examined both the accuracy and response times as subjects performed the CRM task. Accuracy and response time on correct items were modeled first as a function of Old/New Status (Target versus NonTarget items) and Language (Spanish versus English items), and then as a function of Language Switching (Switch versus NonSwitch targets) and Language. Accuracy was assessed with a generalized linear mixed-effects (GLM) model with Subjects and Items (word stimuli) as random effects. Response time was tested with a linear mixed-effects (LME) model with the same random effect structure. To account for the range of frequencies of our stimuli, we included log frequency as a covariate in all models of response time [48]. We report the maximal model that converged, which contained the most allowable number of random slopes [49]. All analyses were conducted using lme4 version 1.1–10 (in R version 3.2.1 [50–51]).

### Microwire recordings

Neural activity was recorded bilaterally from 38 μm diameter microwires implanted in 4 brain areas—hippocampus, amygdala, ventromedial prefrontal cortex, and anterior cingulate cortex (ACC)—as subjects performed the CRM task.

#### Implantation & recording

We used surgical and recording methods, which we have previously described in Valdez et al. [52]. In brief, nine microwires were implanted stereotactically (Medtronic StealthStation) with a 1.5T structural MRI through skull bolts at each recording site protruding from the clinical depth electrodes used to locate epileptogenic focus [53–54]. In the hippocampus, the target for microwire tip placement was the mid-body of the hippocampus; in the amygdala, the target was the center of the amygdala; in the anterior cingulate cortex, the target was the anterior cingulate gyrus, above and behind the genu of the corpus callosum; and, in the ventromedial prefrontal cortex, the target was just below the anterior cingulate gyrus and corpus callosum, in the most anterior portion of the gyrus rectus. Using these techniques, the error in tip placement is estimated to be +/-2mm [55]. While this resolution is insufficient to determine subfields within the hippocampus or nuclei within the amygdala, it is sufficient to allow discovery of neural firing differences between major brain areas and sides of the brain.

Following patients' recovery from surgery, the microwires were connected to headstage amplifiers, which applied a 400x gain to yield eight differential recording channels relative to one ground microwire. Extracellular potentials were low-pass filtered and sampled (16 bits) at 29,412 Hz. Possible action potential events were detected as excursions from baseline in a band-pass filtered signal (300–3000 Hz).

#### Spike sorting

**Fig 2:**
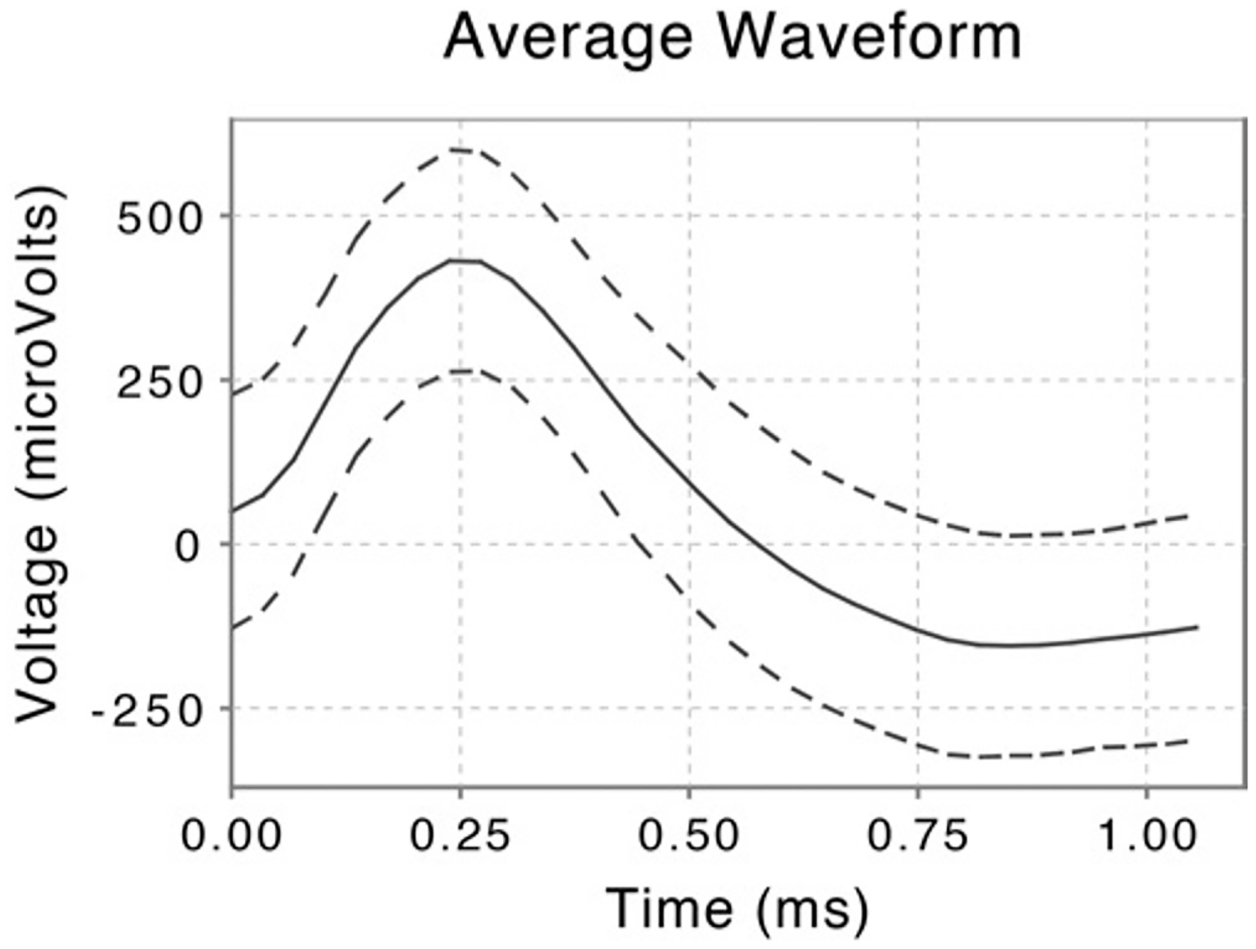
Waveform shape of single-unit activity recorded in the right amygdala. Extracellular potential difference (in microvolts) as a function of time during waveform (in milliseconds) with peak centered at 0.25 milliseconds. Solid line shows average value for all waveforms in cluster and dotted lines show ±1 standard deviation.

Possible action potentials were high-pass filtered from the digitized signal to determine event shape, and all events recorded from individual channels were grouped into sets of similar waveform shape (clusters) with KlustaKwik 1.5 [56], which is based on a modified Celeux-Govaert expectation maximization algorithm [57]. Post-sorting, each cluster was classified as either noise, multi-unit or single-unit activity per the criteria listed in Table 1 of Valdez et al. [52]. Fig 2 illustrates events in a cluster classified as single-unit activity after sorting. See Table 4 for the number of units recorded in each region.

**Table 4:** Number of clusters recorded at each site for each subject.

Area	Side	S26	S37	S40	S49	S50	Total
Amygdala	Left	5/11	0/0	3/2	1/1	1/12	10/26
Amygdala	Right	2/0	2/13	0/0	1/1	3/5	8/19
Anterior Cingulate	Left	0/14	0/0	1/0	2/4	0/0	3/18
Anterior Cingulate	Right	0/6	0/0	0/0	2/8	0/18	2/32
Hippocampus	Left	2/0	0/12	0/0	0/0	1/3	3/15
Hippocampus	Right	2/1	4/10	0/0	0/1	1/11	7/23
Ventromedial PFC	Left	1/7	5/14	0/0	0/0	0/0	6/21
Ventromedial PFC	Right	2/10	0/6	2/0	0/1	1/14	5/31

Values appear separately for single unit recording count/multiunit recording count.

#### Neural response measures

Raw spike counts were tallied in a test period spanning 200–1000 ms after the presentation of each word [58]. The background firing rate was measured during the complementary period prior to the stimulus onset (-200 –-1000 ms). Test period spike counts were then normalized relative to the mean and standard deviation of the background for each cluster.

Our primary interest is in how neural firing may be affected by three types of stimulus-level information, including Old/New Status (Targets, NonTargets), Language Switch Type (Switches, NonSwitches), and the Language in which a word was presented (English, Spanish). To detect such changes in firing, we examined neural activity at two levels. First, we examined whether the average neural firing in a brain area, the average of all the clusters recorded in a brain area, showed significant evidence of changes in average firing as a function of these 3 independent variables and their interactions. Second, we examined whether properties of the distribution of neuronal firing, other than the average, changed as a function of these 3 independent variables. All of these analyses were performed in two ways: those conducted on correct trials only and those conducted on all trials, ignoring accuracy. In the sections that follow, results are reported for the main effect of Old/New Status, the main effect of Language Switch Type, and the interaction of Language and Language Switch Type.

To examine changes in average firing, we aggregated all clusters in a specific brain area and performed a one-sample t-test to determine whether there was a net average change in the normalized firing rates of all clusters in a brain area. A *t*-value greater than zero would indicate on average, a signal present in some brain region is more active for level 1 of a factor (e.g., for Old/New Status, Targets > NonTargets), while a *t*-value less than zero would suggest the opposite pattern (e.g., for Old/New Status, Targets < NonTargets). Because we only maintained specific predictions about hippocampal activity, all *p*-values on pairwise comparisons between factors across the left and right hippocampi were adjusted for two comparisons using a Benjamani-Hochberg correction).

To visualize the overall outcome of this analysis, we plotted the distribution of *t*-values as density histograms. Thus, positively-skewed distributions index increased neural activity for level 1 compared to level 2 of a factor, and negatively-skewed distributions correspond to increased neural activity for level 2 relative to level 1 of a factor.

To determine whether changes in the distribution of firing rates amongst clusters, other than a change in the average firing rate, may have occurred, we produced Q-Q plots to visually distinguish average shifts in the distribution across all clusters versus large effects on a limited subset of items across clusters (see also [58]). Finally, in some cases, we also explored effects within the remaining neuroanatomical areas (i.e., anterior cingulate cortex, amygdala, and ventromedial prefrontal cortex). For these analyses, *p*-values were adjusted for 8 total comparisons to compare across all non-hippocampal regions where we had no a priori predictions about the laterality of activity.

## Results

### Behavioral results

We first examined how well the subjects performed the CRM task and whether their performance depended on whether the word was new or old and which language the word was presented in.

#### Effects of Old/New Status and Language

**Fig 3:**
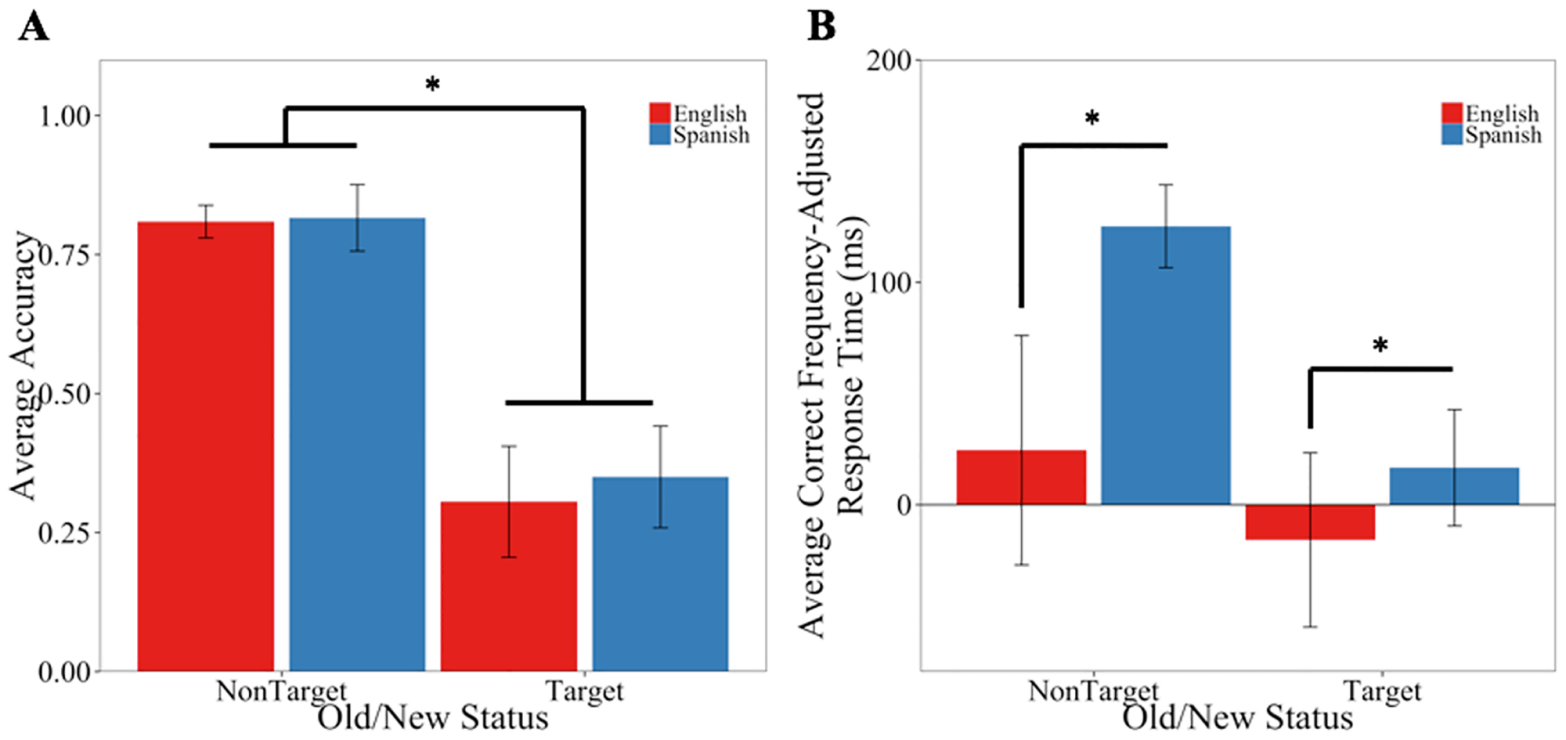
Behavioral patterns as a function of language and Old/New Status. Panel A depicts accuracy and Panel B depicts correct response times adjusted for word frequency, each for Targets and NonTargets split by Language (Spanish and English). * denotes significance at the p<0.05 level.

The maximal GLM model of accuracy included Old/New Status and Language as fixed effects and by-Subject random slopes of both fixed effects and their interaction and by-Item random slopes of Old/New Status. The model revealed a main effect of Old/New Status (*β* = 2.57, *SE* = 0.61, *z* = 4.22, *p*<0.001), such that NonTargets resulted in more accurate responses (*M* = 0.81) compared to Targets (*M* = 0.33; see left panel of Fig 3). There was no significant effect of Language (*β* = -0.25, *SE* = 0.28, *z* = -0.87, *p* = 0.38) and no interaction of these two factors (*β* = -0.04, *SE* = 0.33, *z* = -0.11, *p* = 0.91).

Response times of correct items were evaluated in an LME model with Old/New Status and Language as fixed factors, Log Frequency of each word as a covariate, and by-Item random slopes of Language. We observed main effects of Language (*β* = -77.14, *SE* = 26.79, *t* = -2.88, *p* = 0.004) and Old/New Status (*β* = 62.44, *SE* = 26.94, *t* = 2.32, *p* = 0.02), but no interaction of Old/New Status and Language (*β* = -67.82, *SE* = 51.68, *t* = -1.31, *p* = 0.19). This indicates that correctly recognized Spanish words elicited slower frequency-adjusted response times (*M* = 71ms) compared to their English counterparts (*M* = 4ms), and Targets were processed slower (*M* = 75ms) than NonTargets after frequency was taken into account (*M* = 0ms; see right panel of Fig 3). Overall, Target items led to less accurate processing accompanied by slower response times on correct items relative to NonTarget items. This response time profile is consistent with other CRM data that use a version of the task without language switching [59].

#### Effects of Language Switch Type and Language

**Fig 4:**
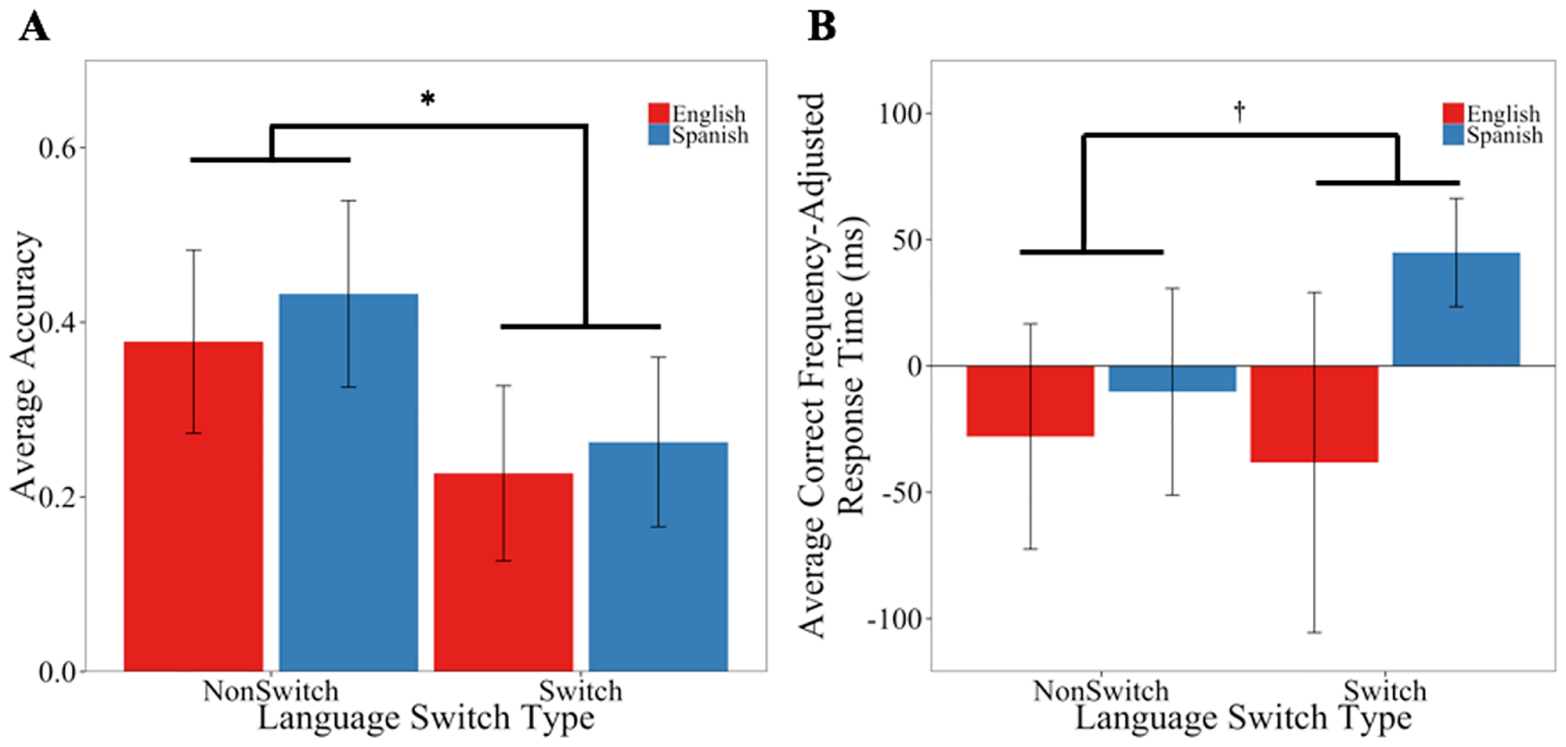
Behavioral patterns as a function of Language Switch Type. Panel A depicts accuracy and Panel B depicts correct response times adjusted for word frequency, each for Switch and NonSwitch cases split by Language (Spanish and English). * denotes significance at the p<0.05 level; ^†^ marginal at the p<0.07 level.

We next examined whether subject’s performance on the CRM task depended on whether the language of word presentation changed between first and second presentations of a word. We modeled accuracy as a function of Language Switch Type and Language with by-Subject and by-Item random slopes of Switch Type. This maximal GLM model revealed a main effect of Language Switch Type (*β* = -1.11, *SE* = 0.43, *z* = -2.62, *p* = 0.009), such that NonSwitch targets were more accurate (*M* = 0.41) than Switch targets (*M* = 0.25; see left panel of Fig 4). There was no significant effect of Language (*β* = 0.27, *SE* = 0.21, *z* = 1.67, *p* = 0.21) and no interaction of Language Switch Type and Language (*β* = -0.02, *SE* = 0.43, *z* = -0.05, *p* = 0.96).

The LME model of correct response times included fixed effects of Language Switch Type and Language, Log Frequency of each word as a covariate, and by-Subject random slopes of Language Switch Type, Language, and their Interaction. This maximal model did not reveal a significant effect of Language Switch Type (*β* = 28.20, *SE* = 59.52, *t* = 0.47, *p* = 0.64). There were also no effects of Language (*β* = -50.38, *SE* = 46.08, *t* = -1.09 *p* = 0.27) and no interaction of Language Switch Type and Language (*β* = -24.51, *SE* = 100.38, *t* = -0.24, *p* = 0.81). To recap, we observed robust effects of language switching in terms of accuracy, but no evidence for a comparable effect on correct response time. Although we have no other bilingual CRM data to call on for comparison, this finding is partially consistent with other work in the domain of cross-linguistic switching that relies on different paradigms to measure performance under switch and non-switch conditions [60–61]. We now turn our attention to measures of neural activity, where we test the hemispheric lateralization hypotheses detailed in the previous section.

### Neural results

#### Effects of Old/New Status

We evaluated the effect of Old/New Status (Target versus NonTarget) on average firing rates using paired *t*-tests that were run over all experimental trials recorded within a brain area. Because there were very few observations at the single-unit level, the results that follow are reported for combined single *and* multiunit activity. We expected the hippocampus to distinguish old and new items, with higher activation for targets relative to non-targets. We first tested this effect in the left and right hippocampi on correct trials.

**Fig 5:**
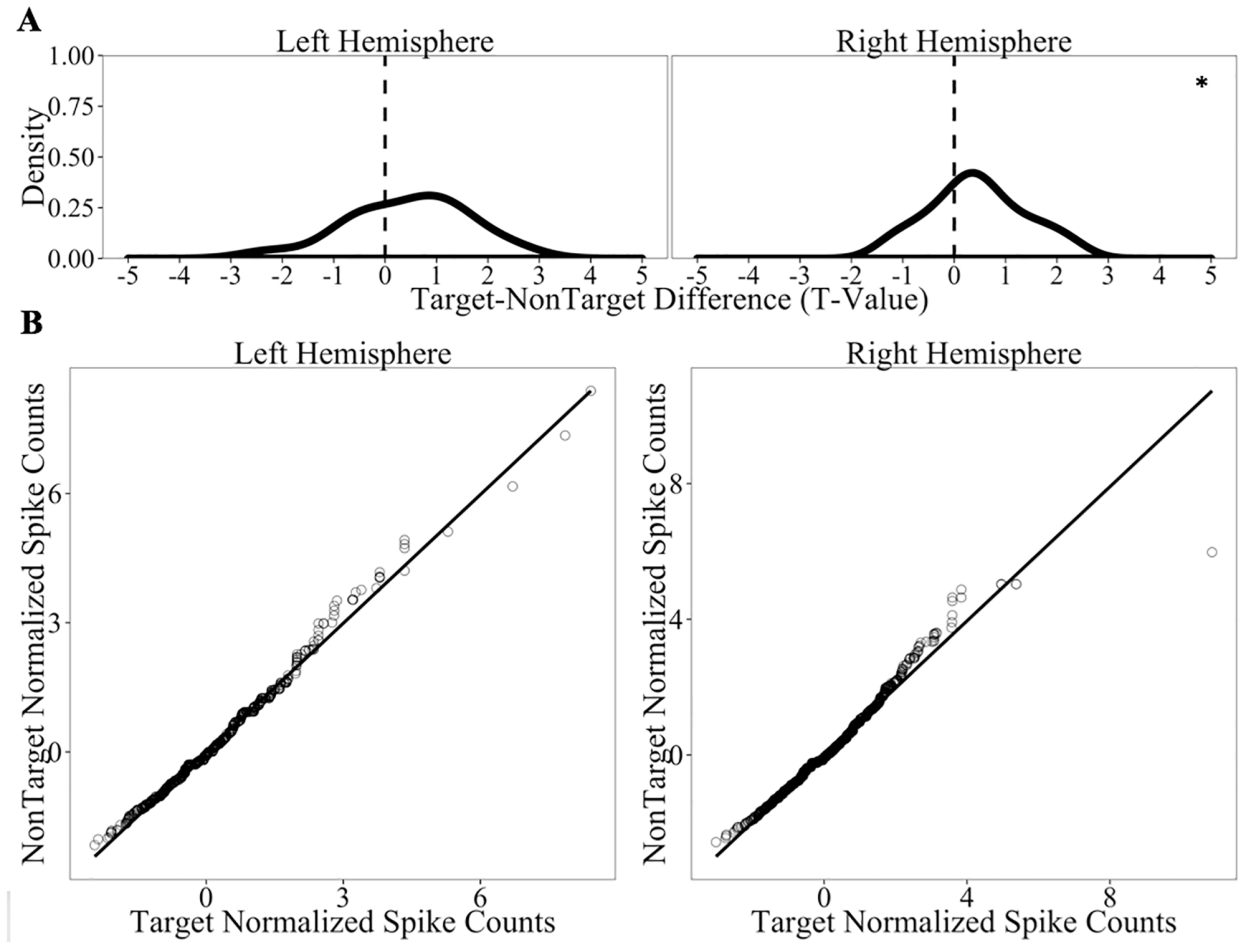
Hippocampal firing rates as a function of Old/New Status on correct trials only. Panel A displays distributions of the *t*-values for Target-NonTarget comparisons of normalized spike counts in all units recorded in the hippocampus. Panel B depicts Q-Q plots comparing the normalized spike count distribution of correct Target trials vs. the distribution of correct NonTarget trials for the left and right hippocampus. * denotes cases when the distribution is different from 0 at the p<0.05 level (all tests corrected for 2 comparisons using Benjamini-Hochberg corrections).

We observed effects of Old/New Status in the right hippocampus (*t*(29) = 2.64, *p*_*adj*_ = 0.03) where there was a greater number of spikes for correct Targets compared to correct NonTargets. No such effect appeared in the left hippocampus (*t*(17) = 1.64, *p*_*adj*_ = 0.24). The *t*-value distribution is positively-skewed for the right hippocampus data (Panel A of Fig 5), illustrating that Targets elicit more activity than NonTargets. Moreover, the data on the Q-Q plot corresponding to the right hippocampus shift away from the reference line (see Panel B of Fig 5), which indicates that the increased activity associated with Targets is not limited to a negligible subset of observations in the right hippocampal units. Left hippocampus activity, on the other hand, revealed a Q-Q plot with virtually no shift or deviations from the reference line, which reflects the proper pattern given a non-significant finding. Interestingly, the effect in right hippocampus weakened when error trials were included (i.e., when we analyze all trials, regardless of performance accuracy; *t* = 2.22, *p* = 0.07). Thus, the hippocampus appears to preferentially register cases when an item is properly recognized a repeat (i.e., hits). Overall, these results are consistent with the prediction that targets should be associated with greater hippocampal firing rates relative to non-targets.

**Fig 6:**
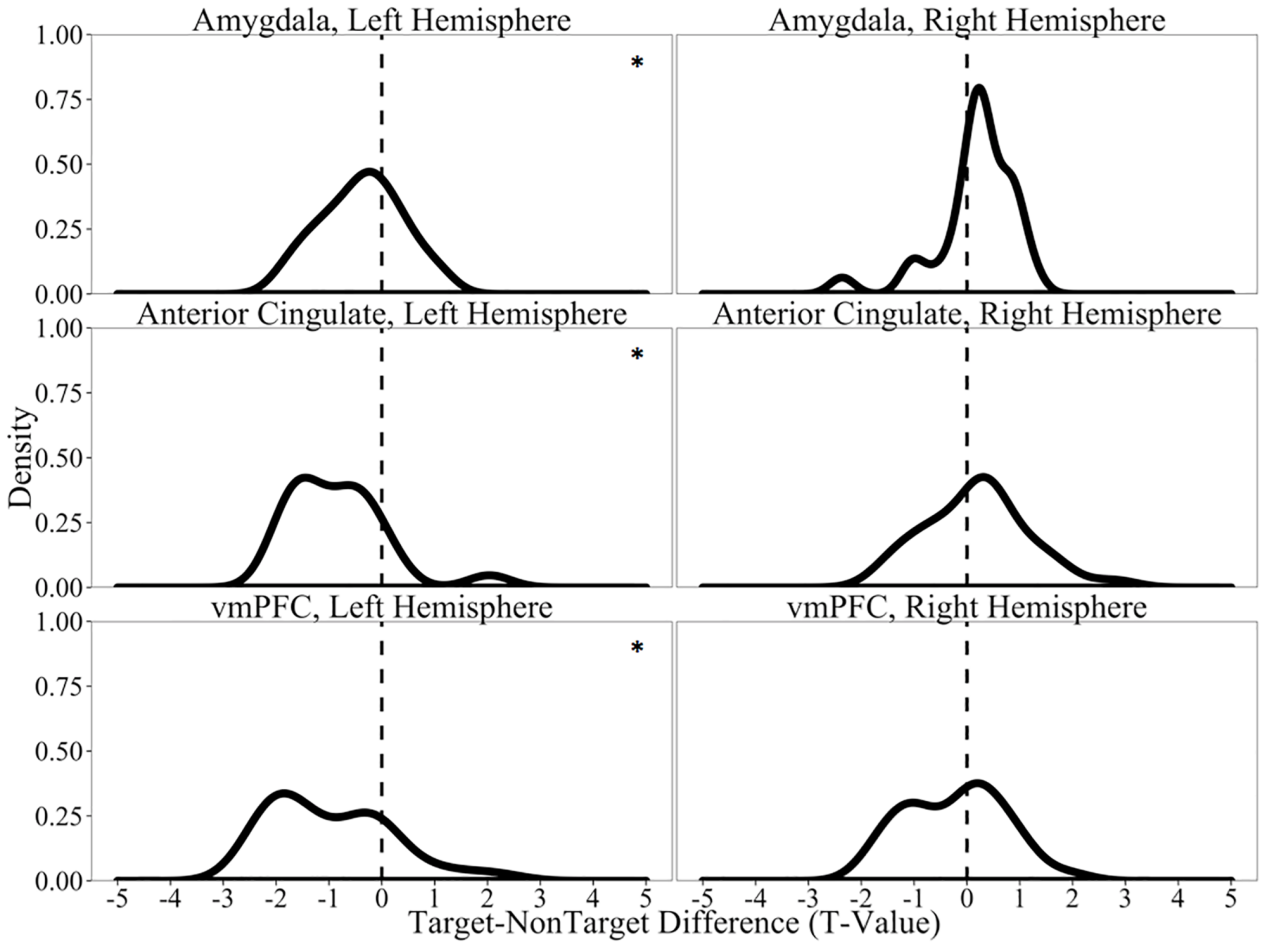
Firing rates in all recorded brain regions as a function of Old/New Status on correct trials only. Distributions of the *t*-values for Target-NonTarget comparisons of normalized spike counts in all units recorded. * denotes cases when the distribution is different from 0 at the p<0.05 level (all tests corrected for 8 comparisons using Benjamini-Hochberg corrections).

In addition to the hippocampus, we also evaluated the effect of Old/New Status in the three remaining brain regions. As can be seen in Fig 6, we found a general trend for increased firing selectively in the left hemisphere for correct NonTargets relative to correct Targets (Amygdala: *t*(35) = -2.93, *p*_*adj*_ = 0.05; Anterior Cingulate: *t*(20) = -4.11, *p*_*adj*_ = 0.004; Ventromedial Prefrontal Cortex: *t*(26) = -4.26, *p*_*adj*_ = 0.002; All right regions: *t*s<1.43, *p*_*adj*_s>0.99). Notably, non-hippocampal brain regions in the left hemisphere generally led to a pattern that did not emerge within the left hippocampus. One possible explanation is that emotional processing (supported by the amygdala and ventromedial prefrontal cortex) and inhibitory control (supported by anterior cingulate cortex) coalesce to support processes involved with novelty detection. Support for this speculation comes from recent neuroimaging findings that indicate that the amygdala is involved in cognitive processing beyond just emotion, including the detection of new information [60]. Specifically, compared to familiar information, new information leads to greater activation in the amygdala [62], as well as greater connectivity between it and sensory regions [63].

#### Effects of Language Switch Type

Next we examined the effect of Language Switch Type on target trials. Similar to the analyses of Old/New Status, we focused on spike counts obtained from both single and multiunit recordings on correct trials. If the data are consistent with the PARLO model detailed above, we predicted reliable, but asymmetrical activation patterns as a function of Language Switch Type in hippocampal neurons. We observed greater activation for correct Switch targets compared to correct NonSwitch targets in the left hippocampus (*t*(17) = 2.68, *p*_*adj*_ = 0.03), but no effect in the right hippocampus (*t*(29) = -1.49, *p*_*adj*_ = 0.29).

**Fig 7:**
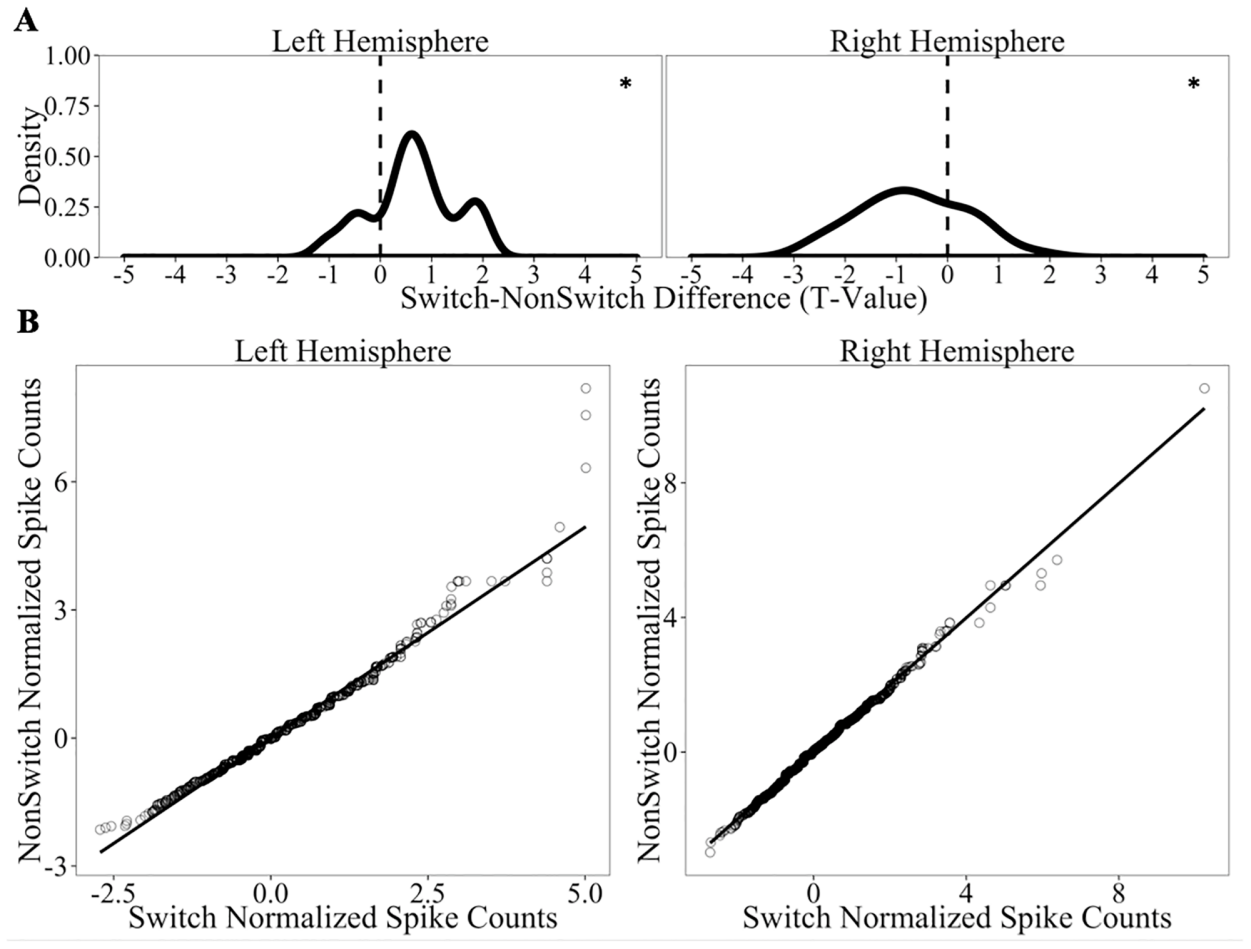
Hippocampal firing rates as a function of Language Switch Type on all trials only (correct and incorrect). Panel A displays distributions of the *t*-values for Switch-NonSwitch comparisons of normalized spike counts in all units recorded in the hippocampus. Panel B depicts Q-Q plots comparing the normalized spike count distribution of correct Switch trials vs. the distribution of correct NonSwitch trials for the left and right hippocampus. * denotes cases when the distribution is different from 0 at the p<0.05 level (all tests corrected for 2 comparisons using Benjamini-Hochberg corrections).

Interestingly, a different profile of results emerged when we considered all trials, regardless of behavioral performance (i.e, correct and incorrect items). In addition to greater firing among Switch targets relative to NonSwitch targets in the left hippocampus (*t*(17) = 3.21, *p*_*adj*_ = 0.01), we observed the reversed effect in the right hippocampus such that there was greater firing among NonSwitch targets relative to Switch targets (*t*(29) = -3.49, *p*_*adj*_<0.003). As Panel A of Fig 7 illustrates, the distribution of unit-level *t*-values is positively-skewed for the left hippocampus and negatively-skewed for the right hippocampus. Furthermore, Panel B of Fig 7 confirms that this pattern is due to the activity of many units; the Q-Q plots of activity in both the left and right hippocampus revealed shifts from the reference lines. As we expand on in the General Discussion, this profile of results is consistent with the PARLO model.

**Fig 8:**
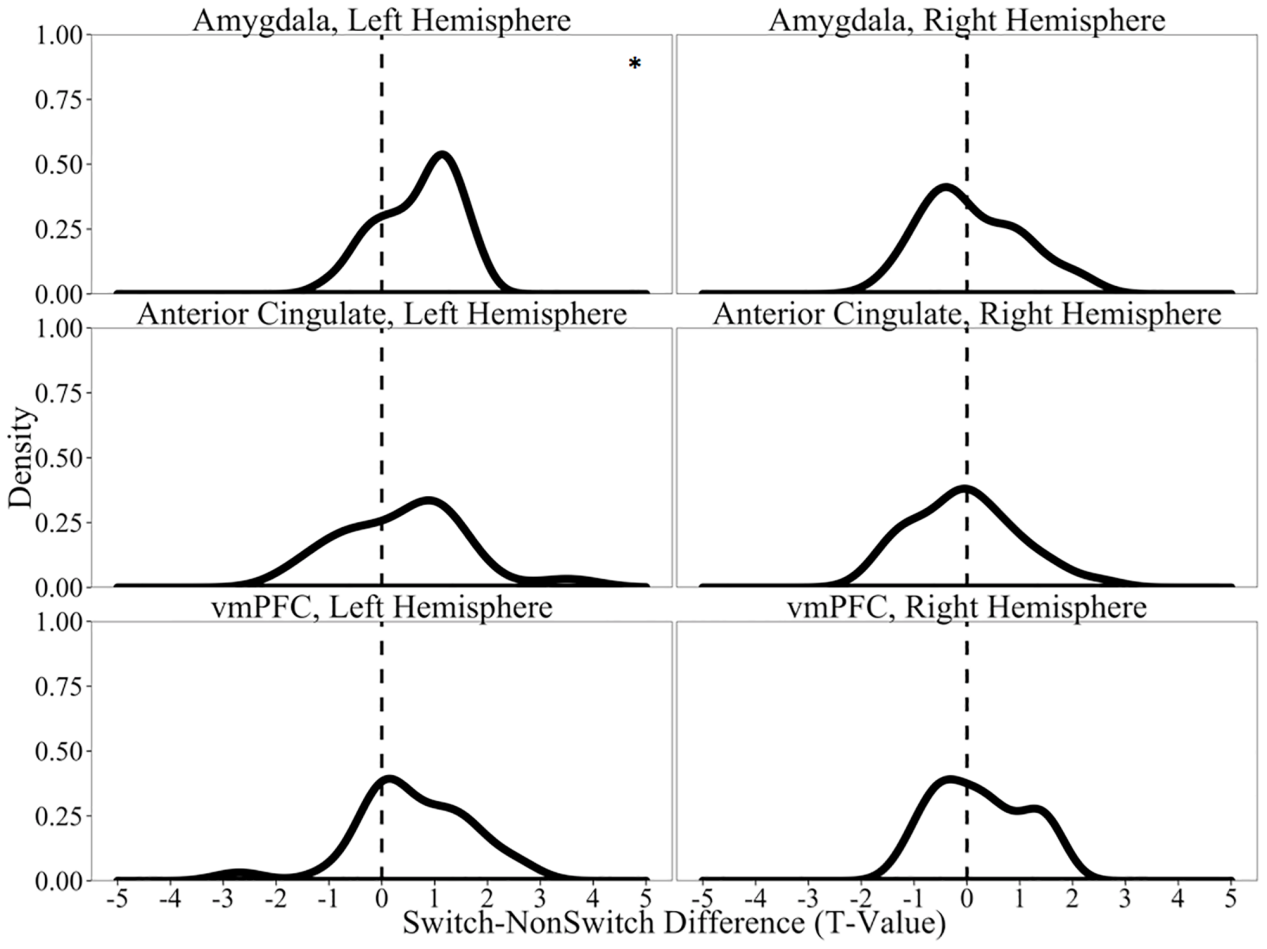
Firing rates in all recorded brain regions as a function of Language Switch Type on all trials (correct and incorrect). Distributions of the *t*-values for Switch-NonSwitch comparisons of normalized spike counts in all units recorded. * denotes cases when the distribution is different from 0 at the p<0.05 level (all tests corrected for 8 comparisons using Benjamini-Hochberg corrections).

In addition to hippocampal activity, we also explored whether spike counts in the remaining brain regions were associated with Language Switch Type. Although we found no significant Language Switch Type effects on correct trials alone (*t*s<2.73, *p*_*adj*_s>0.08), we observed reliable effects on all trials (correct and incorrect) within the left amygdala (*t*(35) = 5.67, *p*_*adj*_<0.001); all other regions: *t*s<2.79, *p*_*adj*_s>0.08). Switch trials were associated with increased activity relative to NonSwitch trials (see Fig 8). Interestingly, this effect appears in an area that is associated with emotional regulation, which indicates that switching across languages within a continuous recognition task may recruit emotional processing resources.

#### Effects of Language and Language Switch Type

In light of extant findings that switching from one’s L1 to one's L2 is especially costly [37,64–65], we tested whether the effects of Language Switch Type within the hippocampus was influenced by the Language (Spanish vs. English) in which a target appeared. Because our subject group was composed entirely of native Spanish speakers, we reasoned that switching from Spanish to English (L1-to-L2) would incur a neural cost larger than that of switching from English to the dominant Spanish lexical item (L2-to-L1). We tested this by conducting separate Language Switch Type analyses for English and Spanish targets, with analyses of English targets reflecting L1-to-L2 processing and those of Spanish targets reflecting L2-to-L1 processing.

**Fig 9:**
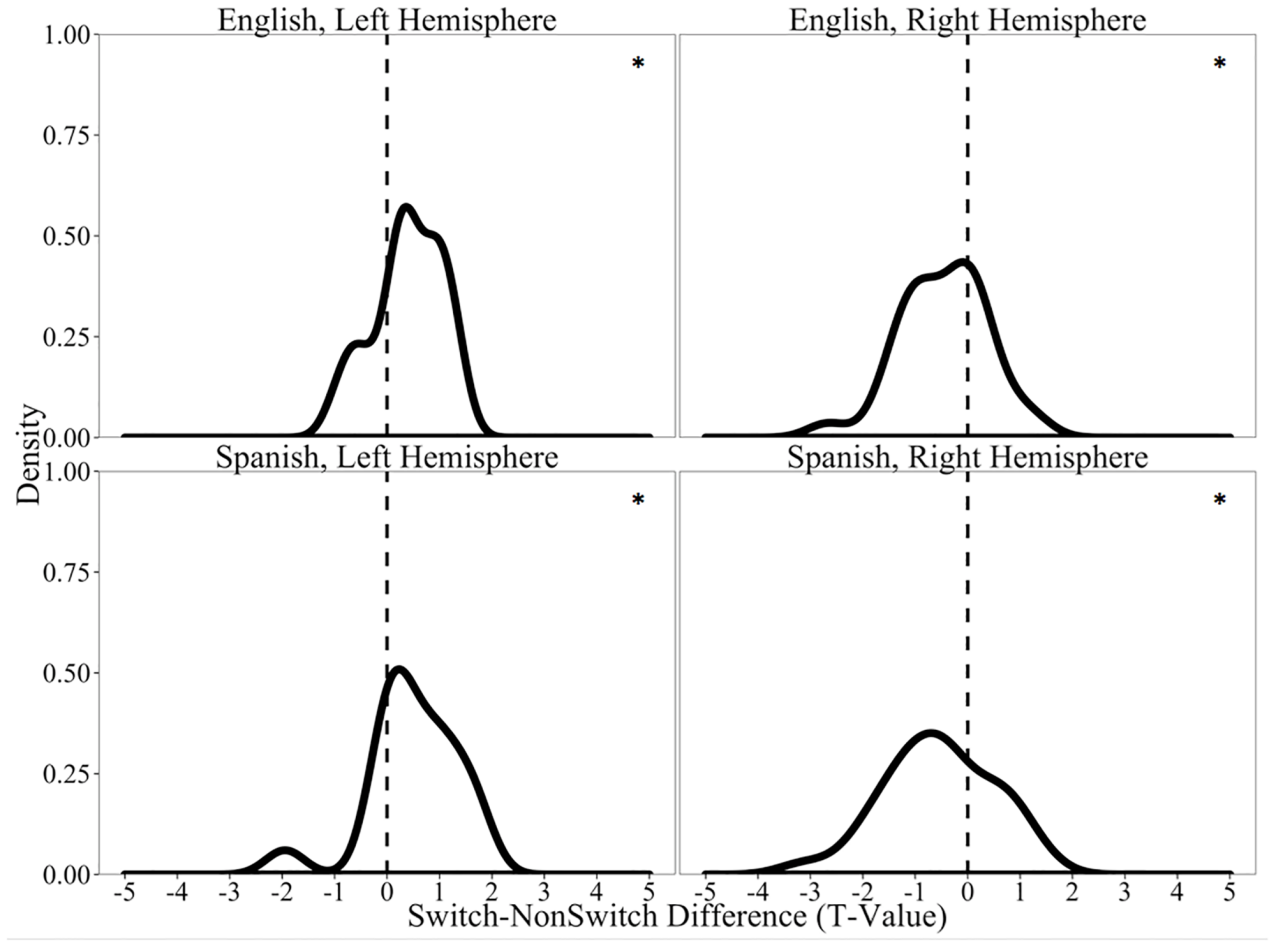
Hippocampal firing rates as a function of Language Switch Type and Language on all trials (correct and incorrect). Panel A displays distributions of the *t*-values for Switch-NonSwitch comparisons among English targets of normalized spike counts in all units recorded in the hippocampus. Panel B displays distributions of the *t*-values for Switch-NonSwitch comparisons among Spanish targets of normalized spike counts in all units recorded in the hippocampus. * denotes cases when the distribution is different from 0 at the p<0.05 level (all tests corrected for 2 comparisons using Benjamini-Hochberg corrections).

In the hippocampus, we observed a significant effect of switching on English targets (i.e., Spanish to English) in both the left (*t*(17) = 2.53, *p*_*adj*_ = 0.04) and right hippocampi (*t*(29) = -2.99, *p*_*adj*_ = 0.01). As can be seen Panel A of Fig 9, the neural activity associated with these switches is positively-skewed in the left hemisphere, indicating more firing for English Switch trials compared to English NonSwitch trials. The opposite pattern appeared in the right hippocampus, where English NonSwitch trials led to more activity than English Switch trials.

Spanish targets resulted in a similar pattern. There was an effect of Language Switch Type in the left (*t*(17) = 2.47, *p*_*adj*_ = 0.05) and right hippocampus (*t*(29) = -2.85, *p*_*adj*_ = 0.02; Panel B of Fig 9). This suggests that the Language Switch Type effects are comparable regardless of the language into which one is switching and regardless of language dominance. This pattern is not consistent with findings that L1-to-L2 switches are more demanding than L2-to-L1 switches. Instead, we report a general cost of switching for Spanish-to-English and English-to-Spanish in Spanish-dominant individuals (though, note that a subset of our participants were fairly balanced between English and Spanish as indicated by the Cloze scores in Table 1).

We also examined the joint effect of Language and Language Switch Type in the remaining brain regions, and found no clear evidence of an effect of switching on English targets in any region (*t*s<2.73, *p*_*adj*_s>0.09). Switch effects of Spanish targets, on the other hand, revealed an effect in the left amygdala (*t*(35) = 5.20, *p*_*adj*_<0.001; all other regions: *t*s<1.61, *p*_*adj*_s>0.10). These results provide the first glimpse of discriminate and direct neuroanatomical resources that support switching into one’s L1 versus L2: Switching into most subjects’ dominant language (Spanish) elicited greater firing in left amygdala, an effect that did not emerge under cases when subjects switched into their non-dominant language (English). This suggests that the Switch effects in left amygdala (see previous section) may have been mediated by cases when subjects must switch from English-to-Spanish. That is, the opposite switch from Spanish-to-English did not elicit a comparable change in neural firing in the left amygdala, where the original switch effect appeared. This builds on functional MRI findings that discriminate cases of switching between linguistic representations from cases of processing input from just one language [66–68]. Finally, alongside the hippocampal findings, our results are compatible with accounts that suggest network-wide support of cross-linguistic switching [18, 67, 69].

## General discussion

The results presented here demonstrate the neural signature, at the single neuron level, of lexical recognition within and across languages in the bilingual brain. A cross-linguistic continuous recognition memory task revealed significant and dissociative effects on neural firing within the left and right hippocampi. Targets that were previously seen in the same language (non-switches) triggered increased activation in the right hippocampus, whereas targets that were previously seen in another language (switches) triggered increased activation in the left hippocampus. This differential cross-hemispheric pattern aligns well with several models of language processing that predict both bilingual and monolingual effects. For example, the PARLO model proposes that the LH supports more abstract, “top-down” processing of semantic content, while the RH places a premium on recognizing input in a veridical, “bottom-up” fashion to enable processing of surface form [15]. Linking this idea to our findings, in order to respond correctly on switch trials, the participant had to access a memory trace for an abstract representation (i.e., a type common to both words), as the veridical representation alone would not yield information that had been previously seen (i.e., two distinct tokens for each word). In the non-switch trials, access to either level of representation could be used to arrive at a correct response. Thus, switching from L1-to-L2 or L2-to-L1 would require the retrieval of semantic information, while non-switch trials, like L1-to-L1 or L2-to-L2 could rely on any information that repeats across items including veridical representations, form, or semantic information.

Outside of the hippocampus, we observed an asymmetry in language switch direction on Switch trials, such that the amygdala was differentially recruited for targets constituting a switch from Spanish-to-English compared to those that were English-to-Spanish switches. Switching from the non-dominant English to the dominant Spanish representation in these relatively dominant Spanish speakers appears to engage the emotional processing resources supported by the amygdala. (Note that early effects of valence on lexical processing have also been reported for monolinguals [70–72].) Interestingly, the lateralized switch effect in hippocampus and the L1-to-L2 amygdala effects are compatible with those reported by Hernandez [34]. One possible interpretation of this pattern stems from recent work in Keysar et al. [73] and Costa et al. [74]. These researchers report a "foreign language effect," whereby bilinguals responding to moral dilemmas and emotion-laden scenarios appear to rely less on emotion-based heuristics in their non-dominant or L2 language compared to when operating in their L1. Costa et al. [74] also presented their participants with emotionally-neutral logic problems, and observed that the foreign language advantage disappeared in their behavioral measures. Although the words presented current CRM task were not particularly emotionally charged, it is possible that even single-word stimuli in one's dominant language are more tightly linked to emotional centers than their translation equivalents in a non-dominant language.

Beyond the amygdala results, it is perhaps surprising that we did not find any compelling evidence that the prefrontal regions from which we recorded (anterior cingulate cortex and ventromedial prefrontal cortex) were sensitive to stimulus presentation in alternating languages. There are however three potential explanations of differences between our findings here and those reported in the neuroimaging literature: differences in the task, differences in the subject population, and differences between measurement techniques.

In terms of the task performed, we note that the bilingual continuous recognition task used here did not require language "switching" as usually conceived or implemented in studies of bilingual language use [75–76]. We note in particular that the lack of observed results in the anterior cingulate cortex (ACC) here contrasts with the results reported by Abutalebi and colleagues [76–77] (see also [78]), in which the ACC played a central role. We believe these differences may be due to the fact that the tasks described in [76–77] are quite different from the task we used here. In [76] paper, the task was to listen to connected discourse that occasionally switched on occasion mid-sentence from one language to another. In the [77] paper, the task was a production task in which participants were required to switch from one language to another. In both of these tasks, inhibition of one language or the other is critical (as Abutalebi at al. [76–77] argue). Assuming that participants in the former task were attempting to construct coherent interpretations of the input, on switch trials they would need to inhibit whichever language the sentence had begun in. In the latter task, participants had to inhibit one language to name a picture in the other. As noted above, inhibition was in fact *not* strictly necessary in our task, even in "switch" trials. In fact, in order to answer correctly 'yes' on switch trials, participants would not need to "switch" at all: they would in fact need to keep both languages active, in order to remember that they had seen some word, in either language, that meant, e.g., RIVER (where the capitalized word stands for the abstract semantic representation of the entity). To answer correctly 'yes' in the "non-switch" trials, they of course would not have to switch (or inhibit another language), either. So, although there may have been some trials on which participants could have consciously or actively inhibited one language in favor of the other, doing so would not have been essential for their successful performance of the task. As such, it is not terribly surprising that we observed no differences in activity in prefrontal regions associated with executive control.

In addition, given that the bilingual CRM task is first and foremost a recognition memory task, it is possible that it was not demanding enough to recruit prefrontal regions or networks associated with language-switching by other researchers (e.g., basal ganglia) [79–81]. For example, the rapid instructed task learning (RITL) task employed in [79] included complex mathematical operations and new instructions at the beginning of each trial, such that each trial was a "novel task."

Differences between patient populations may also account for why we did *not* see differential patterns of activation as a function of switching in prefrontal regions. Recall that our data were recorded from patients with severe epilepsy. Recent neuroimaging results implicating prefrontal regions in language switching come from healthy participants. There are thought to be neuroanatomical differences between patients with epilepsy (as in our population) and healthy individuals [82] (but see [83] for evidence of negligible differences between epileptic and non-epileptic neuron activity). For example, recent work suggests that such patients experience reorganization of language networks that result in great inter-hemispheric activation relative to healthy controls [82]. It is plausible that the current results are a by-product of some gross rewiring of classic language networks, leading to the under-recruitment of prefrontal regions typically observed to be highly active in bilinguals as they move between languages. Future research exploring this speculation would be welcome.

Finally, it is important to note that neuroimaging studies provide an indirect measure of neural activity, typically, changes in the level of the blood oxygen level dependent (BOLD) signal. The exact relationship between this signal and underlying firing of neurons in a given brain area is still unclear [84–87] and may well differ between different brain areas and tasks [88–92]. It is thus perhaps less surprising to see differences between the results reported by direct measures of neural firing, such as reported here, and neuroimaging studies.

### Future directions & concluding remarks

The current results provide a springboard for many areas of future experimentation. First, the sample included only five patients, limiting the power to perform certain multivariate analyses, (e.g., analyses of lag between repeating items, of which we had anywhere from no lag, or an immediate repeat, up to a 32-item lag). Future work could explore the effects of lag and retention in a cross-linguistic task like ours in healthy samples using other methods like co-registration of fMRI and EEG or from non-invasive brain stimulation techniques (like transcranial direct current stimulation or transcranial magnetic stimulation [93]). Second, although we were not able to run an equivalent experiment with monolingual controls (due to the restrictions that come with identifying and testing patients like those who participated in our study), it is possible that a comparable design could be developed to test for similar effects in healthy monolinguals. One example might be a cross-modal running recognition memory task, in which monolinguals judge whether they have seen repeating semantic information that appeared either as pictures or words, irrespective of whether the given stimulus was previously encountered in image or lexical form (cf. [34]). Finally, a wider spread of language dominance, in both directions, among a larger sample of patients would be hugely beneficial in improving both statistical power and the assessment of bilingual models (e.g., RHM) that make explicit predictions with respect to L1-L2 balance.

The present experiment is able to distinguish between theories of language processing in the brains of both monolinguals (e.g., PARLO) and bilinguals (e.g., RHM) at the level of the fundamental computational units of the brain, namely, single neurons. Of particular interest to bilingual language processing is the widespread, bilateral nature of linguistic function in bilinguals. Although adding another language into the mix complicates the study of language in the brain, coordinating, accessing, and generating multiple languages may engage non-linguistic cognitive functions in quantitatively and/or qualitatively different ways than can be observed in monolinguals [34, 40, 94]. For example, compared to monolinguals, bilinguals routinely show more distributed patterns of neural activation [40, 95–96], which often manifests as increases in global bilateral recruitment [39] (see [34] for an example of local bilateral recruitment in hippocampus). For instance, Rüschemeyer and colleagues [97] observed increased activation in the basal ganglia of bilinguals processing language in their L2 compared to in their L1. Indeed, the basal ganglia has been recognized to be integral to higher-order cognitive function [98], including learning and memory [99] and language [100]. Additional evidence suggests that for both mono- and bilinguals, the coordination of lateralized language processes appears to depend on the transmission of information across the corpus callosum [101]. Although we lack a monolingual control group, our results are consistent with this collection of findings, specifically by demonstrating that bilingual processing recruits brain regions across both hemispheres and involves trade-offs between long-term memory and emotional regulation regions within the brain. To close, the present intracranial microwire recordings point toward potentially fruitful avenues and questions for future studies using both this and other intracranial recording methods [13], as well as non-invasive methods to elucidate the cognitive and neural architecture supporting real-time processing efforts in monolinguals and bilinguals.
